# Supplementary material for: 24-hour movement behaviors and cognitive ability in preschool children: A compositional and isotemporal reallocation analysis
Source: PLoS One. 2025 Mar 26;20(3):e0320081. doi: 10.1371/journal.pone.0320081 (PMC11940664; doi:10.1371/journal.pone.0320081)
Supplement: Attachment — Submitted filename: Response_to_Reviewers_auresp_2.docx [file pone.0320081.s004.docx]

**Response Letter**

Dear editors and reviewers,

We are very grateful for your constructive comments and suggestions for our manuscript entitled “***24-hour movement behaviors and cognitive ability in preschool children: A compositional and isotemporal reallocation analysis***” (ID: PONE-D-24-28927). Your comments are very valuable and helpful for improving our manuscript. In the following, the responses to all the comments are provided one by one.

We have tried our best to make all the revisions clear, and we hope that the revised manuscript can satisfy the requirements for publication.

The main revisions in the new manuscript are:

1. Revised the formatting issues in the previous manuscript.

2. To ensure the completeness of Table 4, its position has been adjusted.

3. Unified the names of accelerometers.

4. Improved the process of calculating appropriate sample size.

Sincerely,

Corresponding author.

**Response to the comments of Reviewer #2**

Q1. Standardize the format of accelerometer name, as seen in line 139-140.

Response from the author: The authors are sorry for our careless mistakes. As suggested by the reviewer, accelerometer name has been unified in the revised manuscript, as seen in line 141-142.

Q2. The manuscript title displayed in the submission system is in Chinese. Please modify it.

Response from the author: The authors are sorry for our careless mistakes. As suggested by the reviewer, the manuscript title has been changed to English in the submission system.

**Response to the comments of Reviewer #3**

Q1. This study aims to explore the relationship between 24-hour movement behaviors and cognitive ability, not to estimate the prevalence of a certain disease. Therefore, is the prevalence parameter applicable when estimating the sample size?

Response from the author: Thank you very much for the constructive feedback. This study adopted a reasonable and appropriate sample size calculation method. The previous manuscript imitated Mota et al.'s writing paradigm due to the lack of a good language expression. The authors attempted to find a more reasonable explanation for the calculation process of appropriate sample size based on relevant references of G*power's appropriate sample size calculation method, as seen in line 126-130.

Q2. The manuscript title displayed in the submission system is in Chinese. Please modify it.

Response from the author: The authors are sorry for our careless mistakes. As suggested by the reviewer, the manuscript title has been changed to English in the submission system.

Q3. In the revised content, all the texts are piled together. There is not even a blank line to distinguish the replies to different questions or the replies to different reviewers. It is extremely difficult to read, and I hope it can be improved in the future.

Response from the author: Thank you very much for the constructive feedback. We are so sorry that the authors overlooked the reviewers' reading experience of the revised manuscript in order to try their best to revise the reviewers' comments. We will pay attention in the future.
