# Supplementary material for: 24-hour movement behaviors and cognitive ability in preschool children: A compositional and isotemporal reallocation analysis
Source: PLoS One. 2025 Mar 26;20(3):e0320081. doi: 10.1371/journal.pone.0320081 (PMC11940664; doi:10.1371/journal.pone.0320081)
Supplement: Attachment — Submitted filename: Response to Reviewers.docx [file pone.0320081.s003.docx]

**Response Letter**

Dear editors and reviewers,

We are very grateful for your constructive comments and suggestions for our manuscript entitled “***24-hour movement behaviors and cognitive ability in preschool children: A compositional and isotemporal reallocation analysis***” (ID: PONE-D-24-28927). Your comments are very valuable and helpful for improving our manuscript. In the following, the responses to all the comments are provided one by one.

We have tried our best to make all the revisions clear, and we hope that the revised manuscript can satisfy the requirements for publication.

The main revisions in the new manuscript are:

1. The relevant literature on the impact of different sleep parameters and types of prolonged sitting on cognitive development in preschool children has been added.

2. The estimation of minimum sample size and recruitment flowchart have been updated.

3. The errors in the statistical paragraph have been corrected and relevant supporting references have been added.

4. The methods of sleep measurement and cognitive measurement were explained.

Sincerely,

Corresponding author.

**Response to the comments of Co-EIC and Associate Editor**

Co-EIC: When submitting your revision, we need you to address these additional requirements.
Q1. Please ensure that your manuscript meets PLOS ONE's style requirements, including those for file naming.

Response from the author: Thank you for your kind suggestion. The style of the revised manuscript and the naming of the files have been changed according to the requirements of PLOS ONE.

Q2. Please note that PLOS ONE has specific guidelines on code sharing for submissions in which author-generated code underpins the findings in the manuscript. In these cases, we expect all author-generated code to be made available without restrictions upon publication of the work.

Response from the author: Thank you very much for the editor's attention and recognition of our research work. We fully understand your journal's requirements for code sharing. Due to our laboratory's policies or confidentiality agreements, research codes are not publicly available. The corresponding code can be provided to qualified researchers upon request by the corresponding author.

Q3. Please note that funding information should not appear in any section or other areas of your manuscript. We will only publish funding information present in the Funding Statement section of the online submission form. Please remove any funding-related text from the manuscript.

Response from the author: Thank you for your kind suggestion. In the revised manuscript, the funding-related text has been removed from the manuscript.

Q4. Thank you for stating the following financial disclosure: “Hunan Provincial Education Science Planning Project Base Project, grant number XJK23AJD055”.

Please state what role the funders took in the study. If the funders had no role, please state: "The funders had no role in study design, data collection and analysis, decision to publish, or preparation of the manuscript." If this statement is not correct you must amend it as needed. Please include this amended Role of Funder statement in your cover letter; we will change the online submission form on your behalf.

Response from the author: Funders played an important role in article quality monitoring, publication decisions, manuscript review, and editing.

Q5. We note that you have indicated that there are restrictions to data sharing for this study. PLOS only allows data to be available upon request if there are legal or ethical restrictions on sharing data publicly.

Response from the author: Thank you very much for the editor's attention and recognition of our research work. We fully understand your journal's requirements for data sharing. Due to our laboratory's policies or confidentiality agreements, data supporting research results are not publicly available. The corresponding code can be provided to qualified researchers upon request by the corresponding author.

Data owner organization:

Organization Name: Hunan Research Centre for Excellence in Fitness

Email: wangshiqiang@hut.edu.cn

Q6. Your ethics statement should only appear in the Methods section of your manuscript. If your ethics statement is written in any section besides the Methods, please move it to the Methods section and delete it from any other section. Please ensure that your ethics statement is included in your manuscript, as the ethics statement entered into the online submission form will not be published alongside your manuscript.

Response from the author: Thank you very much for the constructive feedback. The ethical statement in this article has been removed from the section outside the methods and added to the methods section.

**Response to the comments of Reviewer #1**

Q1. Please rephrase “This is due to the subpar quality of the studies and the limited accumulation of research evidence.” Or provide evidence for why the studies are “subpar” – what does this mean?

Response from the author: The authors apologize for confusing the reviewer. The author wants to express that due to different existing research schemes, it is still unclear which PA intensity is more beneficial for cognitive ability.

The author has removed the original wording and rephrased “due to differences between the PA protocol and the results reported in previous research reports, it is currently unclear which elements of PA are more beneficial for the cognitive abilities of preschool children”.

Q2. For the statement “Moreover, different sleep parameters also have differences in the development of cognitive abilities in early children [11]” please provide some context of what these sleep parameters are. Will they be explored in this study?

Response from the author: As suggested by the reviewer, the authors have researched and added more literatures to support the differential effects of different sleep parameters on cognitive development in preschool children, and they are referred as [7] and [17] in the revised manuscript, as seen in line 58-60. In addition, this study only measured the sleep duration of preschool children. Therefore, this study only discusses the impact of sleep duration on cognitive development in preschool children.

Q3. “Similarly, the association between SB and cognitive development in early children should also vary depending on the type of sedentary behavior [12].” Can you please provide some examples here.

Response from the author: As suggested by the reviewer, the authors have researched and added more literatures to support the differential effects of different SB types on cognitive development in preschool children, and they are referred as [9] and [18] in the revised manuscript, as seen in line 64-54.

Q4. “The advantage of this analysis method is that it fully corresponds to the requirements of the behavioral guidelines, with satisfaction marked as 1 and dissatisfaction marked as 0, resulting in a clear and intuitive presentation of the data.” This is confusing to me. Does satisfaction mean “compliance with the guidelines” and how does this work when there are multiple guidelines each for different behaviours within the 24 h day, i.e., it’s possible to meet some guidelines (sleep and PA), and not meet others (screen time).

Response from the author: The authors apologize for confusing the reviewer. The author intends to highlight the advantages of using component data multiple linear regression by examining the shortcomings of the multiple mixed linear regression research method. Therefore, the author changed the originally unclear wording to address the shortcomings of the multiple linear regression research method. as seen in line 82-87.

Q5. “A study from low-income areas in Brazil found a positive correlation between 24-hour movement behaviors and EF in pre-school children” This doesn’t make sense – it’s not possible to have a positive correlation between all 24-h MBs and an outcome? Do you mean there was an association between the composition and EF?

Response from the author: The authors are sorry for our careless mistakes. What the author wants to express is that there is a correlation between 24-hour MBs and EF, and the positive has been removed, as seen in line 92-94.

Q6. Please refine the Aims
Aim 1: i think this is to evaluate the associations between the overall 24-h MB composition and outcomes?

Response from the author: Thank you for your kind suggestion. This is the most core content explored in this study. Due to the author's wording, it caused confusion for the reviewers. The authors deeply apologize and have made changes in the manuscript.

Q7. Aim 2: please reword to avoid inference of directionality which is not possible with cross-sectional data (“difference” rather than “change”)

Response from the author: Thank you for your insightful suggestion. Isotemporal Substitution Paradigm is based on the correlation between the proportion of existing 24-hour movement behavior time and the cognitive ability of preschool children, simulating two movement behaviors to replace each other at the same time, and predicting the expected theoretical changes in cognitive ability of preschool children. Therefore, the authors believe that changes are reasonable.

Q8. Please ensure all methods are written in past tense.

Response from the author: Thank you for your insightful suggestion. The authors have changed all the grammar in the research methodology section to the past tense.

Q9. There are grammatical errors in sentence structure which will require editorial attention.

Response from the author: Thank you for your insightful suggestion. A careful proof-reading has been conducted for the revised manuscript to improve the language and grammar.

Q10. Re the statement : “Forty-two households still need to provide written consent.” Please clarify that these participants have therefore been excluded from this analysis.

Response from the author: Thank you very much for the constructive feedback. 42 invited participants were excluded from this analysis due to their guardians not providing written consent.

Q11. How were day time naps recorded/dealt with?

Response from the author: Thank you for your insightful question. Before the start of this study, the researchers established WeChat groups with parents and teachers. The day time nap on weekdays is recorded by the teacher. However, it is relatively difficult for the teacher to record the bedtime and wake-up times of multiple children during their time in the kindergarten. Therefore, the teacher took photos of each child bedtime and wake-up times during the day and checked in the group. Researchers recorded the nap time of young children during the day by uploading photos from teachers. Parents record the nap time of young children during weekends and daytime, as seen in line 160-162.

Q12. Before creating ilrs, you need to check if there are any zero values in any of the compositional parts, or any NA values. All zeros need to be replaced with small values and all NA values must be removed before applying the ilr transformation. Please specify if/how many zeros were replaced in your methods.

Response from the author: Thank you for your insightful question. During the data collection process, there were 27 preschool children with missing 24-hour movement behavior data. Therefore, the data of 27 preschool children has been excluded before applying the ilr transformation.

Q13. Please clarify this sentence: “Applied the isometric log-ratio (ILR) transformation to compositional data, converting each composition (e.g., duration of SP, SB, LPA, and MVPA) into a logarithmic ratio relative to the geometric mean of other compositions [28].” To “: “An isometric log-ratio (ILR) transformation was applied [28].” It does not matter what type of ilr transformation is used for this model, so it is not necessary to describe it.

Response from the author: Thank you for your insightful suggestion. As suggested by the reviewer, the author simplified "Applied the isometric log ratio (ILR) transformation to compositional data, converting each composition (e.g. duration of SP, SB, LPA, and MVPA) into a logarithmic ratio relative to the geometric mean of other compositions [28]" and simplified it to "An isometric log ratio (ILR) transformation was applied [28]", as seen in line 234.

Q14. How did you account for potential clustering at the child care centre level?

Response from the author: Thank you for your insightful question. The preschool education in the urban area of Zhuzhou City is divided into four sections. This study strategically selected one kindergarten from each of the four districts, in order to ensure a representative sample among different education departments in the city, as seen in line 118-119.

Q15. Line 205: “d represents the number of compositions,” Please correct the terminology throughout this section. The “composition” refers to all the behaviours or compositional parts (sleep, sed, LPA and MVPA) together. You are using it here to refer to a single part. Due to multiple errors in this paragraph, I suggest omitting this technical/statistical paragraph and refer to the statistical papers instead. This is especially pertinent as you are using the compositional isotemporal substitution model for which the type of ilr transformation is irrelevant (you will get the same results regardless of the sequential binary partition used to make the ilrs).

Response from the author: Thank you for your insightful suggestion. As suggested by the reviewer, the authors omitted this statistical paragraph and referred to relevant statistical papers, as seen in line 245.

Q16. “There is no significant difference between 224 arithmetic percentage and component percentage.” This was not tested by a statistical test, so please reword.

Response from the author: Thank you for your insightful suggestion. The authors omitted this sentence.

Q17. The pair-wise log-ratio presents co-dependency between behaviours, not “correlation” or “Association” – please reword the results around this.

Response from the author: Thank you very much for the constructive feedback. The author changed "correlation" or "association" to "co-dependent", as seen in line 265-267.

Q18. Line 230 onwards – please also provide the multivariate test statistic for the overall composition. You may need to orient the table vertically to make it fit.

Response from the author: Thank you very much for the constructive feedback. The multivariate test statistics for the overall composition are shown in Table 3, as seen in page 13.

Q19. Line 245: “Based on the 95% confidence interval (CI), compositional isotemporal substitution was carried out for cognitive ability outcome measures.” Please remove the words “Based on the 95% confidence interval (CI),” so that this sentence makes sense.

Response from the author: Thank you very much for the constructive feedback. As suggested by the reviewer, the authors have deleted “Based on the 95% confidence interval (CI)”.

Q20. Results and Discussion and Conclusion: throughout: when talking about reallocation results, please reword “increases” ,“decreases” and “changes” to “higher”, “lower” and “differences” to avoid causal/directional language.

Response from the author: Thank you very much for the constructive feedback. When reallocating the results, it is to compare the expected theoretical results with the results of the study on the impact of the proportion of 24-hour movement behavior time on the cognitive ability of preschool children. Therefore, the authors believe that the use of words such as "increases," "decreases," and "changes" are reasonable.

Q21. “The dose-response (Figures 1-3) result shows a significant asymmetry in pre-school children's total” please avoid the word “significant” as it implies a statistical test has been conducted to show that there is asymmetry.

Response from the author: Thank you very much for the constructive feedback. The authors removed the word “significant” and changed “The dose-response (Figs 1-3) result shows a significant asymmetry in pre-school children's total” to “The dose-response (Figs 1-3) results revealed that there was asymmetry in the changes of FIQ in preschool children when MVPA and other movement behaviors were replaced with each other”, as seen in line 396-397.

**Response to the comments of Reviewer #2**

Q1. The research involved 191 preschool children from Zhuzhou city in a cross-sectional study. ls this sample size sufficient to support the conclusions drawn in the study? And please ask the author to unify the sampling locations in the abstract and methods, so that readers can have a clearer understanding of the data analysis process in the manuscript and improve the readability of the article.

Response from the author: Thank you very much for the constructive feedback. The authors estimated the minimum number of participants required for the study using G * Power (3.1.9). In addition, the sampling locations in the abstract and methods have been unified.

Q2. In the results section, SP, SB, LPA, and MVPA are almost all abbreviated throughout the manuscript, while in the descriptive statistics, the sleep period uses the full name. Please standardize the format for the author.

Response from the author: Thank you very much for the constructive feedback. The authors changed all “SP” abbreviations to “sleep”.

Q3. Please explain what the "-30-" in the title of the figure means and how it affects its readability. If necessary, please optimize the title of the figure.

Response from the author: Thank you for your insightful question. “−30- to 30-min” represents the range of reallocating time from -30 minutes to 30 minutes. The authors have optimized the title of the figure, as seen in line 313-314, 317-318 and 321-322.

Q4. Please improve your English expression.

Response from the author: Thank you very much for the constructive feedback. We have tried our best to polish the language in the revised manuscript.

**Response to the comments of Reviewer #3**

Q1. Line 55-56, “previous studies have found…”, citations are needed.

Response from the author: Thank you for your insightful suggestion. In the revised manuscript, it has been added as the references [19], as seen in line 71.

Q2. Line 59-60, this sentence is too wordy, please simplify it.

Response from the author: Thank you very much for the constructive feedback. The authors have simplified the sentence, as seen in line 73-75.

Q3. Line 73, citations are needed to support your idea.

Response from the author: Thank you for your insightful suggestion. In the revised manuscript, it has been added as the references [24], as seen in line 88.

Q4. In the methodology, there should be a flowchart of subject recruitment and eventual inclusion in the statistical analysis. Also, please report the number of boys and girls separately.

Response from the author: Thank you for your insightful suggestion. As suggested by the reviewer, the authors have added a participant recruitment flowchart (Fig 1) and reported the number of boys and girls, as seen in line 133.

Q5. The sample size of this study is relatively small. Can it achieve sufficient statistical power? Please add the process of sample size estimation.

Response from the author: Thank you for your insightful suggestion. The authors estimated the minimum number of participants required for the study using G * Power (3.1.9), as seen in line 124-126.

Q6. Line 124-126, citations are needed.

Response from the author: Thank you for your insightful suggestion. In the revised manuscript, it has been added as the references [30] and [31], as seen in line 148-150.

Q7. The activity characteristics of preschool children are short duration and high frequency, so the sampling interval is generally 1s. Why did this study use 15s?

Response from the author: Thank you for your insightful question. The first reason for choosing 15s in this study is that the sampling interval for establishing the cut-off point for preschool children is 15s, so this study should also use a 15s sampling interval. The second is based on the selection of previous literature. A review of methods for measuring physical activity in preschool children shows that the proportion of using a 15s sampling interval is as high as using a 60s sampling interval.

Q8. The main exposure factor of this study is 24-hour movement behavior, including physical activity and sedentary time measured by objective methods, and sleep time measured by subjective methods. Usually the sum of the results measured by the two methods will not be exactly equal to 24 hours. How did the author process the data so that the time of physical activity + sedentary time + sleep time is equal to 24 hours?

Response from the author: Thank you for your insightful suggestion. Firstly, the authors think that the sleep time of preschool children refers to their bedtime and wake-up time. Secondly, during the daytime nap in the kindergarten, the teacher took photos to check in and sent them to the WeChat group. The researchers recorded the children's daytime sleeping time on weekdays, while the children's nighttime sleeping time and weekend daytime nap time were both recorded by their parents. The research stipulates that parents should remove the accelerometer and take photos to check in and send them to the WeChat group after the child goes to bed. When he woke up in the morning, his parents helped him get a good accelerometer and took photos in the WeChat group to check in. Finally, when the researchers processed the data in the software, they compared the recorded sleep time of the children frame by frame. Therefore, the time of physical activity + sedentary time + sleep time is equal to 24 hours.

Q9. How many examiners take part in the IQ test for preschool children? If there are multiple examiners, how can we ensure the consistency of the results from different examiners?

Response from the author: Thank you for your insightful question. Due to the cognitive test being divided into two parts. Therefore, this study invited two professionally trained researchers, each responsible for evaluating a section to ensure consistency in the evaluation criteria for each section. Ultimately ensuring the consistency of the overall score, as seen in line 174.

Q10. Line 170, citations are needed.

Response from the author: Thank you for your insightful suggestion. In the revised manuscript, it has been added as the references [14] and [38], as seen in line 204.

Q11. In addition to age and gender, other factors that affect preschool children's cognitive abilities should also be considered, such as BMI, parents' education, income, etc. In particular, maternal education level, previous research has shown that it has an extremely significant impact on preschool children's cognition.

Response from the author: Thank you for your insightful suggestion. The authors agree with the reviewer that other factors that affect preschool children's cognitive abilities should also be considered, such as BMI, parents' education, income, etc. However, this is also a limitation of this study, and future research must take these factors into consideration.

Q12. Line 209, please cite references to support your explanation of isotemporal substitution model.

Response from the author: Thank you for your king suggestion. In the revised manuscript, it has been added as the references [41], as seen in line 248.

Q13. Line 320-321, please express your idea in a scientific report way.

Response from the author: Thank you for your king suggestion. The authors intended to express that PA intensity may be an important factor affecting the cognitive development of preschool children. When considering the PA intensity of preschool children, MVPA performs stronger in different cognitive abilities compared to LPA, as seen in line 359-362.

Q14. Why does the content of advantage appear before the title of “Strengths and limitations”?

Response from the author: Thank you for your king suggestion. The authors have removed the word “Strengths”, as seen in line 419.

Thanks to the professional comments again that point out the above problems. The authors hope these explanations would answer your doubts.
